# Is there a role for immunotherapy in HER2-positive breast cancer?

**DOI:** 10.1038/s41523-018-0072-8

**Published:** 2018-08-15

**Authors:** Esther Holgado, Jose Perez-Garcia, Maria Gion, Javier Cortes

**Affiliations:** 1 Ramon y Cajal University Hospital, Ctra, Colmenar Viejo km. 9,100, 28034 Madrid, Spain; 2 IOB Institute of Oncology, Quiron Group, Madrid, Spain; 3 IOB Institute of Oncology, Quiron Hospital, Plaça d’Alfonso Comín 5, 08023 Barcelona, Spain; 4 Medica Scientia Innovation Research (MedSIR), Barcelona, Spain; 5 Vall d´Hebron Institute of Oncology, Centro Cellex, Calle Natzaret 115-117, 08035 Barcelona, Spain

## Abstract

Although the prognosis and prediction of therapeutic benefit for breast cancer patients overexpressing the human epidermal growth factor receptor 2 (HER2) have dramatically changed with the administration of anti-HER2-targeted therapies, HER2-positive metastatic breast cancer is still an incurable disease. Thus, new and better therapeutic options are urgently needed. Among them, are the agents stemming from the field of immunology that have been the focus of impressive scientific progress and new therapeutic opportunities seem to emerge “every day” in a variety of tumor types.

## Introduction

Although breast cancer is considered a moderately immunogenic tumor,^1,2^ deeper research into its molecular subtypes has shown striking heterogeneity in the frequency and diversity of somatic mutations between tumors. In fact, the tumor mutational burden and the presence of tumor infiltrating lymphocytes (TILs), which clearly correlate with clinical outcomes,^3–5^ are higher among triple-negative (TNBC) and human epidermal growth factor receptor 2 positive (HER2+) breast carcinomas than in ER+ tumors.^6^ Based on the observed responsiveness of highly mutated tumors with high infiltration of immune cells to immunotherapeuticals, HER2+ remains an optimal setting to explore the efficacy of cancer immunotherapy (CIT).

## TILs and HER2-positive breast cancer

There is a growing body of evidence about the biological function of infiltrating immune cells in controlling tumor growth and progression. In fact, the tumor cell compartment and the surrounding stromal microenvironment is a subject of continuous modification across the different stages of progression. In this scenario, the relationship between both compartments is a complex network of interactions that involves the production of tumor-related chemokines that attract the immune cells to the host anti-tumor response.

Several studies have shown that TILs are associated with favorable long-term prognosis and better response to therapy in HER2-positive breast cancer.^7^ In the early setting, a clinical study with 232 HER2+ BC patients (FinHer trial) were randomized to receive adjuvant chemotherapy with or without Trastuzumab. A 10% increment of the TIL score was associated with an 18% reduction in the relative risk of distant recurrence, which reflects the association of TILs with increased disease-free survival when trastuzumab was added to chemotherapy.^8^ These findings were confirmed by the NSABP B-31 clinical study of trastuzumab plus adjuvant chemotherapy for HER2+ BC patients.^9^ In this study, the group of patients with high expression of TIL-associated genes showed a higher benefit from treatment with trastuzumab, demonstrating the role of TILs as predictors of disease-free survival (HR: 0.06; 95% CI: 0.01–0.47, *p* = 0.007). By contrast, in the N9831 clinical study, the addition of anti-HER2 therapy was not superior in patients with a high level of TILs (HR: 2.43; 95% CI: 0.58–10.22, *p* = 0.22).^10^ This non-significant association was partially explained by authors as a consequence of the low rate of events found in the high TIL subgroup. Of interest, a deeper analysis of the N9831 trial studying TILs with an immune gene signature (IGS) showed a 63% benefit in the relapse-free survival of the IGS-enriched patients treated with trastuzumab compared to those who received chemotherapy alone (HR: 0.36; 95% CI: 0.23–0.56, *p* = 0.001).^11^

The impact of TILs to predict the benefit of anti-HER2 therapies was assessed in more than 2000 patients in the neoadjuvant setting in different clinical trials: NeoALTTO, Cher-Lob, CALGB 40601, GeparSixto, and the Neosphere trials.^12–16^ In summary, a high presence of TILs in the stromal compartment of HER2+ primary tumors prior to the anti-HER2 therapy was associated with a higher rate of pathological complete responses (pCR).

The role of TILs in HER2-positive metastatic breast cancer has been much less studied and much more data is needed. The highest body of evidence comes from the CLEOPATRA study, where patients were randomized to receive docetaxel and trastuzumab with either pertuzumab or placebo. Two interesting findings were observed; on one hand, each 10% increase in TILs was significantly associated with longer overall survival (HR: 0.89; 95% CI: 0.83–0.96, *p* = 0.0014); and on the other hand, freshly metastatic tumor samples had significant lower TIL values than did the archival samples (10% vs. 15%, respectively).^17^

All the above suggest that anti-HER2 therapies play a significant role in modulating the stromal immune compartment and opens up important questions for patient management. Thus, below, we summarize the evidence of anti-HER2 therapeutics through the modulation of the immune response in BC patients.

## Immunotherapy in HER2-positive breast cancer

Although trastuzumab, a humanized IgG1 monoclonal antibody (mAb) specific against ERBB2, was initially developed to inhibit the trophic support provided by the HER2 protein kinase receptor to the malignant cells,^18^ an immune-based mechanism of action has also been observed. BC patients have shown a robust infiltration of lymphoid cells in the tumor after treatment with trastuzumab.^19^ Indeed, a pilot study of neo-adjuvant trastuzumab with 18 BC patients suggested that the anticancer effects are not only mediated by the direct inhibition of the HER2 signaling pathway but also through the induction of antibody-dependent cellular cytotoxicity (ADCC). In fact, the constant fragment (Fc) in the IgG1 of Trastuzumab can be recognized by Fc-receptors (FcR) in NK cells and macrophages and these cell populations were amplified in peripheral blood samples in 83% of the patients.^20^ Moreover, there is some evidence which suggests that specific FcR genotypes are associated with improved progression-free survival (PFS) in response to Trastuzumab.^21^

An appealing strategy to improve the efficacy of CIT through the induction of immunogenic cell death has emerged.^22^ The foundation of the immunogenic death is the release of intracellular molecules, such as ATP and calreticulin among others, which may act as signaling molecules to recruit immune cells of the innate response to subsequently activate the adaptive immune response. Interestingly, trastuzumab may also promote immunogenic cell death, resulting in the uptake of breast cancer-associated antigens (BCAA) by dendritic cells (DCs), presentation and elicitation of a CD8+-specific immune response against the BC cells that harbor BCAA. In the NeoPHOEBE trial, neoadjuvant trastuzumab was combined with either the pan-PI3K inhibitor Buparlisib or placebo for 6 weeks, followed by the addition of paclitaxel. Of great interest, the tumor samples obtained just after 15 days of therapy were infiltrated with a significantly higher number of TILs compared to the TIL score in the baseline pre-treatment biopsies, a fact that was highly correlated with pCR.^23^

The ADCC effect of trastuzumab as an additive mechanism of action to targeting HER2+ BC cells might be suggested by the EGF104900 clinical trial of lapatinib ± trastuzumab in metastatic BC patients that progressed to trastuzumab.^24^ This study shows the superiority of trastuzumab plus lapatinib combination over lapatinib alone in patients who progressed on trastuzumab-based therapy in terms of PFS (HR: 0.74; 95% CI: 0.58–0.94, *p* = 0.011) and OS (HR: 0.74; 95% CI: 0.57–0.97, *p* = 0.026). The potential role of ADCC exerted by the IgG1 Fc could explain the striking activity of trastuzumab in this study when HER2 was nicely blocked with lapatinib. In fact, lapatinib blocks HER2 signaling by stabilizing the expression of the HER2 protein at the membrane of BC cells, which increases the levels of the HER2 protein that might be available for binding to trastuzumab to recruit FcR on the mononuclear immune cells. Thus, ADCC might be further activated upon the combination of these two anti-HER2 agents.

Pertuzumab, a humanized IgG1 mAb targeting HER2 in a different domain compared to trastuzumab, prevents HER2 homodimerization and heterodimerization.^25^ The addition of pertuzumab to trastuzumab therapy has been studied in vitro.^26^ Although both trastuzumab and pertuzumab effectively activate ADCC against human BC cells with equal potency, there was no observed synergistic induction of ADCC with the combination, so the clear synergism and high activity that these two monoclonal antibodies have may not be a consequence of a more potent immune activity.

Based on the induction of ADCC exerted by the anti-HER2 mAbs, preclinical studies have addressed the potential synergistic effect of either trastuzumab or trastuzumab emtansine (T-DM1) plus mAbs against the cytotoxic T lymphocyte-associated antigen 4 (CTLA4) and the programmed cell death protein 1 (PD1),^27,28^ demonstrating a strong lymphocytic induction against BC cells. Not surprisingly, the combination of T-DM1 with immune checkpoint inhibitors (anti PD-1 and anti CTLA-4) resulted in curative responses in animal models despite primary resistance to trastuzumab. Based on these data, the combining PD-1 and CTLA-4 inhibitors with anti-HER2+ therapies are in clinical development. In the PANACEA phase Ib/II, the anti-PD-1 pembrolizumab in combination with trastuzumab was explored in metastatic HER2+ BC patients who had progressed to trastuzumab.^29^ Objective response rates (ORR) of 15.2% in the 40 PD-L1-positive BC patients and 0% in the 12 PD-L1-negative patient cohort were reported. Although the vast majority of patients had low numbers of TILs in the metastatic niche, those with TILs above 5% in the tumor sample were associated with an ORR of 39% vs. 5% in those patients with lower TILs (<5%).

Another two optimized mAbs against HER2 are also being studied in the clinical setting. Margetuximab is a next generation Fc-optimized mAb that targets HER2 with a Fc region that increases its ability to mediate Fc domain-dependent ADCC. Engineered to block HER2 signaling with an increased affinity for CD16A polymorphisms while a decreased affinity for the inhibitory FcγRIIB (CD16B) receptor on immune effector cells, such as DCs, NK, monocytes, and macrophages,^30^ margetuximab has showed single-agent activity against heavily pretreated HER2+ BC patients in a phase I trial. A randomized phase III trial in patients with HER2+ BC that progressed to a prior anti-HER-2 therapy is ongoing (SOPHIA, NCT02492711). MCLA-128 is a full length IgG1 bispecific antibody with enhanced ADCC activity that targets HER2 and HER3 to overcome HER3-mediated resistance under HER2-/EGFR-targeted therapies. The preclinical results of the comparative efficacy of MCLA-128 vs. Trastuzumab + Pertuzumab in in vivo models reported a higher antitumor efficacy of MCLA-128. Thus, a first-in-human phase 1/2 study in solid tumors is ongoing (NCT02912949).

Based on the findings mentioned above, the stimulating immune effect of an anti-HER2 tyrosine kinase inhibitor, such as lapatinib to potentially increase ADCC against BCAA HER2 protein to induce recruitment and/or expansion of TILs, has led to an emergent field of novel strategies to further exploit immunotherapy in HER2-positive breast cancer. Very promising approaches that combine the immunogenic properties of HER2 with immune agonizts, such as 4-1BB (CD137), biespecific T cell engagers, IL-2 fusion proteins to expand TILs populations, vaccination with HER2 peptides to increase its immunogenic potential, and adoptive therapies with chimeric antigen receptor T (CAR-T) cells against HER2 arise to the arena of BC therapy. The future results of the randomized phase 2 study AVIATOR (NCT03414658) of Utomilumab to target 4-1BB in combination with avelumab and trastuzumab in patients that progressed to prior trastuzumab and pertuzumab will set the expectations of complex immunotherapy combinations in HER2 BC. The increased recruitment of TILs and their intratumoral expansion expected with the combination of IL-2 variant targeting fibroblast activation protein-alpha in combination with trastuzumab in phase I (NCT02627274) and 41BB/HER2-directed bispecific CD3-T cell engagers, NCT03330561 and NCT02829372, respectively, represent with CAR-T cells more examples of the growing interest of immunotherapy in BC.

## Conclusion and final remarks

Although a significant increase in knowledge in the immune-oncology field has been achieved, the role of immunotherapy in breast cancer is clearly behind other tumor types and the clinical development is still in its early days. In HER2+ BC, TILs are clinically relevant and represent pre-existing anti-tumor immunity which is not only predictive of response but also has a prognostic relevance.

Based on preliminary results of immune checkpoint blockade from early phase clinical trials in HER2+ BC patients, combinations of these agents with anti-HER2 therapies are of great interest for the medical community.

However, some aspects will help us to better position immunotherapy in HER2-positive breast cancer in the future. First, although the current evidence supports the selection of PD-L1 patients, it is too early to anticipate a lack of efficacy in PD-L1-negative patients; second, the role that chemotherapy might play in this setting is still not well known; third, if immunotherapy might work better in earlier lines of therapy is still controversial in HER2-postive breast cancer; fourth, dealing with heterogeneity will help us to better understand the exact role of immunology in breast cancer in general, and HER2-positive BC in particular; and fifth, how to integrate PD1/PD-L1 expression and TILs in clinical research and which is the optimal way of measuring them are not well understood yet.

One thing is clear: HER2-positive metastatic breast cancer is still an incurable disease and more therapies are urgently needed. The dream of curing our patients helps us to work harder and to find more drugs with different properties and mechanisms of action. If immunotherapy is one of these tools will be known in the very near future.
